# Innovative approach in the treatment of comminuted proximal phalanx fractures in horses based on biomechanical modelling

**DOI:** 10.1038/s41598-025-95577-8

**Published:** 2025-04-19

**Authors:** Bernard Turek, Krzysztof Jankowski, Marek Pawlikowski, Tomasz Jasiński, Małgorzata Domino

**Affiliations:** 1 Department of Large Animal Diseases and Clinic, Institute of Veterinary Medicine, Warsaw University of Life Sciences (WULS – SGGW), Nowoursynowska 166, 02-787 Warszawa, Poland; 2 Institute of Mechanics and Printing, Warsaw University of Technology, ul. Narbutta 85, 02-524 Warszawa, Poland

**Keywords:** Third metacarpal bone, External fixator, Screw configuration, Finite element method, Nonlinear viscoelasticity, Equine, Biomedical engineering, Fracture repair

## Abstract

Proximal phalanx (P1) fractures in horses are relatively common, and present significant treatment challenges, especially when the fractures are comminuted or infected. An innovative treatment approach includes attaching an external fixator to the third metacarpal bone (MC III), the healthy bone above fracture, to offload the injured P1 and protect it from load–bearing forces, particularly during post–surgical standing up. This study aims to develop the favourable mathematical and numerical models for screws configuration in this external fixator. Nine configurations (I-IX), varying in screw alignment and number, were investigated based on the experimental data from computed tomography and simulations of compression tests. Cortical and trabecular tissues were modelled as a nonlinear viscoelastic continuum, with material constants identified through uniaxial compression and stress relaxation tests. The best attachment of the external fixator was analysed in terms of stresses and strains in both trabecular and cortical bone, as well as stresses in screws. Configuration II (1 diaphysis screw, 4 distal metaphysis screws at 7°) and III (1 diaphysis screw, 4 distal metaphysis screws at 14°) were identified as mostly biomechanically favourable. This external stabilization approach could potentially reduce the rate of post–surgical failure often leading to horse euthanasia.

## Introduction

Proximal phalanx (P1) fractures in horses are a relatively common clinical problem in equine orthopaedics, constituting a significant portion of fracture cases occurring in equine athletes^1–3^. A fracture is evaluated to determine whether it is simple, multiple (complex), or comminuted, whether it is unicortical or bicortical, and whether there is articular involvement. This initial assessment allows for the establishment of a treatment protocol and prognosis for the horse. A simple fracture consists only two fracture fragments, while complex and comminuted fractures involve three or more fragments. A comminuted fracture, in particular, is characterised by the bone being broken into several small pieces, typically resulting from high–force injuries^1–6^. Complex and comminuted fractures of the P1 most often occur in racehorses working at high speeds^2^ and sport horses, particularly show jumpers, due to the overload experienced upon landing after jump^3^. These fractures can also be seen in horses at pasture across all disciplines^4^. In racehorses and show jumpers, the forelimbs are affected twice as often as the hindlimbs, with no significant preference for the left or right side^5,6^.

Despite significant advances in veterinary medicine over the past decades^7^, the treatment of complex and comminuted P1 fractures remains challenging, with outcomes often proving unsatisfactory^8,9^. Many P1 fractures are so severe that internal fixation with screws alone, or even in combination with plates, is unlikely to succeed. In cases involving open and infected fractures, the patient’s life may be at risk^10^. One may note that most simple P1 fractures are treated surgically, often with good outcomes, enabling horses to return to their previous level of work^3,8,11,12^. Longitudinal proximal sagittal fractures, for example, are typically treated with orthopaedic metal screws that provide sufficient strength, allowing the horse to stand after the surgery without compromising the fixation^8,11,12^. However, the situation is completely different for longitudinal frontal fractures^10,12^, where the more pronounced transverse component makes fixation with screws or plates mechanically untenable^6,13,14^. In such cases, fixation with metal screws does not provide the same strength as with sagittal fractures^15,16^ due to the absence of a biarticular strut of bone bridging the proximal interphalangeal and the metacarpophalangeal joints^13,14^. In complex fracture cases, while screws may secure bony fragments, they do not provide sufficient strength to restore a biarticular strut of bone capable of supporting the horse standing post–surgery^6,13,14^. This inadequacy is critical to the failure of screw fixation alone and can hinder proper fracture healing.

The primary challenge in treating complex and comminuted P1 fractures with screws is the failure of fixation when the horse stands up immediately after surgery or during the recovery period^10,12^. Fixation failure occurs due to the inadequate strength of the fixation and the excessive forces transmitted through the bone fragments during weight–bearing on the limb, often resulting in fatal consequence^13–16^. Another significant challenge is ensuring patient comfort post–surgery and the need to completely offload the affected limb, which leads to overloading of the contralateral limb^17,18^. Given that supporting limb overload for < 24 h is a risk factors for laminitis^19^, prolonged overloading can result in secondary postoperative complications, such supporting limb laminitis^17,18^. In both scenarios, the worst outcome may necessitate humane euthanasia of the horse^17,18,20^, while the best–case scenario often involves poor fracture healing, resulting in the horse losing its previous abilities post–treatment^12,21^. To address this issue and improve treatment outcomes, it is essential to either reduce or completely eliminate the forces acting on the fixated bone fragments. While external stabilization, such as using a fiberglass cast after osteosynthesis, can slightly reduces the risk of fixation failure, it does not fully solve the problem^8,22^. A fiberglass cast provides stabilization but does not sufficiently relieve forces acting on the fracture^15,23^. Furthermore, 49% of horses treated with fiberglass cast showed various types of complications^24^. Therefore, introducing a new type of external stabilization could potentially reduce the rate of post–surgical failure, which often leads to serious consequences, including euthanasia^10,17,18,20,25^.

In this study, we propose the computational model of the use of an external fixator to treat P1 fractures in horses. Here, we present our custom–designed external fixator constructed for treatment the third metacarpal bone (MC III) fractures^26,27^ and now adapted specifically for treating P1 fractures in horses. The primary function of the external fixator is to protect the fractured bone from load–bearing forces, particularly during standing up, by offloading the fractured P1, transferring the forces to the affected limb’s healthy MC III, and thereby relieving the contralateral limb to prevent supporting limb laminitis. The idea of this fixator is based on the external fixators commonly used in human orthopedics^28^; however, its detailed construction is adjusted to the specific nature of horses. It is designed to be attached to the proximally located healthy bone, the MC III, using approximately five 6–mm screws, causing minimal damage to healthy bone tissue. This method offers several advantages, including a short assembly and disassembly time and minimal tissue injury damage. This novel orthopedic device is designed to accommodate the unique anatomical and behavioral characteristics of horses^26,27^. It is easy to install, and the time required for fixation is significantly shorter than that the from plate implantation. Additionally, the screws can be removed without the need for general anaesthesia. One major challenge in treating fractures with metal plates in horses is the scarcity of soft tissue (skin and subcutaneous tissue). This often makes skin closure difficult post–surgery^29,30^. However, this issue is eliminated with an external fixator, as the main structure is positioned outside the limb. Due to the lack of direct contact between the stabilizer structure and the bone (except at the screw heads), there are no issues with the blood supply to the bone^26,27^. Unlike plates^29,30^, the external fixator can be used to treat open fractures, which are often contaminated and infected^26^. The risk of infection at the screw sites can be mitigated through appropriate bandage and antimicrobial treatment^23^. Fixating the external device to the healthy MC III is advantageous, as it avoids the risk of insufficient support for the screws, which might occur if they were placed in the relatively small, fractured P1 bone^22^.

This study aims to develop the mathematical and numerical models of the bone-fixator system with emphasis on the bone–screws interaction to assess the effectiveness of an external fixator in P1 fracture treatment. Specifically, the biomechanical behaviour of equine cortical and trabecular bone was represented using a non-linear viscoelastic constitutive model to determine the most biomechanically favourable screw configuration for attaching an external fixator to the MC III. The material parameters for this model were identified based on experimental testing of equine bone samples.

## Methods

### Computed tomography imaging and bone sampling

This computational modelling was developed based on the computed tomography (CT) imaging and bone samples properties collected from a 5 years old Thoroughbred gelding. The reason behind euthanasia was unrelated to the current study. Therefore, we confirm that no animal was anesthetized/euthanized during the study. After euthanasia, the right front limb was isolated at the level of the carpometacarpal joint. The experiments on cadaver limbs have been reported. The research using the samples collected postmortem does not fall under the legislation for the protection of animals used for scientific purposes (national decree-law Dz. U. 2015 poz. 266 and EU directive 2010-63-EU). No ethical approval was needed.

The isolated front limb were imaged using a 64–slice CT scanner (Revolution CT, GE Healthcare, USA). The imaging was performed using a helical scan type with a current of 275 mA, voltage of 120 kV, gantry rotation of 0.08/s/HE+, table travel of 39.4 mm/rotation, pitch of 0.984:1, and slice thickness of 0.625 mm. The scan length was adjusted between the carpometacarpal joint and the tip of the hoof. Imaging was obtained at a 16–bit quality resolution and saved in DICOM format using the AW workstation (GE Healthcare, USA) and Volume Share software version 7 (GE Healthcare, USA). The CT imaging were performed at the Large Animal Clinic, at the Institute of Veterinary Medicine at the Warsaw University of Life Sciences.

After the CT imaging, the samples of cortical and trabecular bone were collected from the MC III diaphysis and the distal MC III metaphysis, respectively. Bone samples were then cut out using the saw blade machine. The following parameters of the cutting process were applied: (i) saw blade shaft speed *n* = 3300 rpm, (ii) saw blade feed f = 0.17 mm/s, (iii) blade appropriate to cut materials of hardness 50–400 HV. Two samples of cortical bone were cut out from the dorsal part of MC III diaphysis. Two samples of trabecular bone were cut out from the right and left part of the distal MC III metaphysis at the level of the right and the condyle of the MC III. Trabecular bone samples were cut out concerning the natural direction of the trabeculae, which is along the direction of the principal stresses. The dimensions of cortical bone samples were 7 × 7 × 12 mm, and those of the trabecular bone samples were 10 × 10 × 14 mm. All samples were stored at the temperature − 30ºC enclosed in double plastic bags to prevent them from drying.

### Mechanical tests

Two types of tests were performed on both trabecular and cortical bone samples, i.e. uniaxial compression tests and stress relaxation tests. The experiments were conducted on hydraulic MTS Bionix System machine (MTS Systems, USA) at the Institute of Mechanics and Printing, Warsaw University of Technology. The bone samples were loaded in the direction of the longitudinal axis of MC III bone. Before each test the samples were kept for an hour at room temperature 293 K to allow them to thaw. The uniaxial compression tests (the loading and unloading phases) were conducted at the strain rate $\:\dot{\epsilon\:}=0.01\:{\text{m}\text{i}\text{n}}^{-1}$. The maximal compressive forces in the test, to which the samples were compressed, for the trabecular bone sample was 3000 N, and for the cortical bone sample 7000 N. During the stress relaxation tests, samples of both bone tissues were compressed to the strain value of 0.01 within 5 s. The process of stress relaxation was registered for 1200 s.

### Formulation of the constitutive model

In the present study the trabecular and cortical bone tissues were modelled as a non–linear viscoelastic continuum. This approach has been successfully used in modelling the behaviour of human trabecular bone^31^ and bovine cortical bone^32^. In non–linear viscoelasticity, the constitutive equation is formulated as a convolution of strain–dependent elastic stress $\:{\mathbf{S}}^{\mathbf{s}}\left(\lambda\:\right)$ and a time–dependent function $\:g\left(t\right)$:

1 $$\:\mathbf{S}\left(\lambda\:,t\right)=\:{\mathbf{S}}^{\mathbf{s}}\left(\lambda\:\right)*g\left(t\right),\:\:$$

where $\:\mathbf{S}$ – second Piola–Kirchhoff stress tensor, $\:{\mathbf{S}}^{\mathbf{s}}$ – elastic second Piola–Kirchhoff stress tensor, $\:\lambda\:$ is stretch ratio along the loading direction, $\:t$ represents time. Equation (1) can be written as:

2 $$\:\mathbf{S}\left(t\right)=\:{g}_{\infty\:}{\mathbf{S}}^{\mathbf{s}}\left(t\right)+\sum\:_{i=1}^{n}\underset{0}{\overset{t}{\int\:}}{g}_{i}\cdot\:{e}^{-\frac{t-s}{{\tau\:}_{i}}}\frac{\partial\:{\mathbf{S}}^{\mathbf{s}}\left(s\right)}{\partial\:s}ds.\:$$

The function *g*(*t*) may be defined by means of a Prony series as:

3 $$\:g\left(t\right)={g}_{\infty\:}+\sum\:_{i=1}^{n}{g}_{i}\cdot\:{e}^{-\frac{t}{{\tau\:}_{i}}},$$

where: $\:{\tau\:}_{i}$ – relaxation times, $\:{g}_{i}$ – viscoelastic constants,

4 $$\:{g}_{\infty\:}=1-\sum\:_{i=1}^{n}{g}_{i}.$$

The variable $\:s$ in Eq. (2) represents the historical time. To calculate $\:\mathbf{S}\left(t\right)$ the strain history has to be known. Relaxation times and viscoelastic constants are identified using data from experiments.

The elastic part of the second Piola–Kirchhoff stress is calculated using of Eq. (5):

5 $$\:{S}_{ij}^{s}=2\partial\:\varPsi\:/\partial\:{C}_{ij},\:\:\:\:\:\:\:\:\:\:$$

where $\:{C}_{ij}$, (i, j = 1, 2, 3) are components of the right Cauchy deformation tensor **C**.

The strain energy function $\:\varPsi\:$ can be written in general form^33^:

6 $$\:\varPsi\:={\varPsi\:}_{iso}+{\varPsi\:}_{v},\:\:$$

where $\:{\varPsi\:}_{iso}$ represents the isochoric part of strain energy function and $\:{\varPsi\:}_{v}$ represent volumetric part. In this constitutive equation $\:{\varPsi\:}_{iso}$ takes the form of the hyperelastic Mooney–Rivlin model^34^ which for two–parameter model is:

7 $$\:{W}_{izo}={c}_{10}\left({\stackrel{-}{I}}_{1}-3\right)+{c}_{01}\left({\stackrel{-}{I}}_{2}-3\right),$$

and five–parameter model is:

8 $$\:{W}_{izo}={c}_{10}\left({\stackrel{-}{I}}_{1}-3\right)+{c}_{01}\left({\stackrel{-}{I}}_{2}-3\right)+{{c}_{20}\left({\stackrel{-}{I}}_{1}-3\right)}^{2}+{c}_{11}\left({\stackrel{-}{I}}_{1}-3\right)\left({\stackrel{-}{I}}_{2}-3\right)+{{c}_{02}\left({\stackrel{-}{I}}_{2}-3\right)\:}^{2}\:,$$

where $\:{\stackrel{-}{I}}_{1}$ and $\:{\stackrel{-}{I}}_{2}$are the first and second invariants of tensor $\:\stackrel{-}{\mathbf{C}}={J}^{-2/3}\mathbf{C}$, $\:J$ is the determinant of the deformation gradient tensor, $\:{c}_{10}$, $\:\:{c}_{01}$, $\:\:{c}_{11},\:\:{c}_{20}$ and $\:{c}_{02}\:$are hyperelastic constants which are identified on the basis of experimental data.

The volumetric strain energy function $\:{\varPsi\:}_{v}$ takes the form^33^:

9 $$\:{\varPsi\:}_{v}=\frac{1}{D}{\left(J-1\right)}^{2}.\:\:\:\:$$

The constant $\:D$ is the compressibility parameter, which for incompressible materials has to be small and is determined from the experimental test.

By means of algorithm used in Ref.^35^, stress in Eq. (2) can be computed using the formula:

10 $$\:\mathbf{S}\left(t+1\right)={g}_{{\infty\:}}{\mathbf{S}}^{\mathbf{s}}\left(t+1\right)+\sum\:_{\text{i}=1}^{\text{n}}\left({\text{e}}^{\frac{-\varDelta\:t}{{\tau\:}_{i}}}{\cdot\:\mathbf{Q}}_{\mathbf{i}}\left(\text{t}\right)+{g}_{i}\frac{1-{\text{e}}^{\frac{-\varDelta\:t}{{\tau\:}_{i}}}}{\frac{\varDelta\:t}{{\tau\:}_{i}}}\left({\mathbf{S}}^{\mathbf{s}}\left(t+1\right)-{\mathbf{S}}^{\mathbf{s}}\left(t\right)\right)\right).\:$$

In Eq. (10), $\:\varDelta\:t$ represents the time increment, $\:{\mathbf{Q}}_{\mathbf{i}}\left(\text{t}\right)$ is the stress at the previous time step, $\:n$ indicates a number of relaxation times $\:{\tau\:}_{i}$ and viscoelastic constants $\:{g}_{i}\:(i=1,\:\dots\:,\:n)$. Because initial stress and strain in the material are known, the stress at time *t > 0* can be calculated.

### The constants calibration

The constants calibration process was divided into two stages. In the first stage, the number $\:n$ and values of relaxation times were determined using data from stress relaxation tests performed on the trabecular and cortical bone samples taking advantage of the algorithm described in details in Ref.^35^. The algorithm is based on iterative calibration. The relaxation times $\:{\tau\:}_{i}\:(i=1,\dots\:,\:n)$ were calibrated by fitting the curve described by Eq. (10) to the relaxation part of the curve obtained from the experiment. The process of the calibration was conducted in Matlab using author’s script.

In the second stage, the hyperelastic constants, as well as viscoelastic constants $\:{g}_{i}\:(i=1,\:\dots\:,\:n)$, were calibrated using an indirect method based on finite element analyses. These constants were calibrated by fitting the curves obtained from simulations of uniaxial compression tests to the corresponding experimental curves. To complete the task, the constitutive equation was implemented into ANSYS Workbench 2021 R1 (Ansys Inc., USA) using two material model options, i.e. the Mooney–Rivlin hyperelastic model (purely elastic response) and Prony Shear Relaxation series (viscoelastic properties). During the process of the calibration two–parameter and five–parameter Mooney–Rivlin models were considered.

**Fig. 1:**
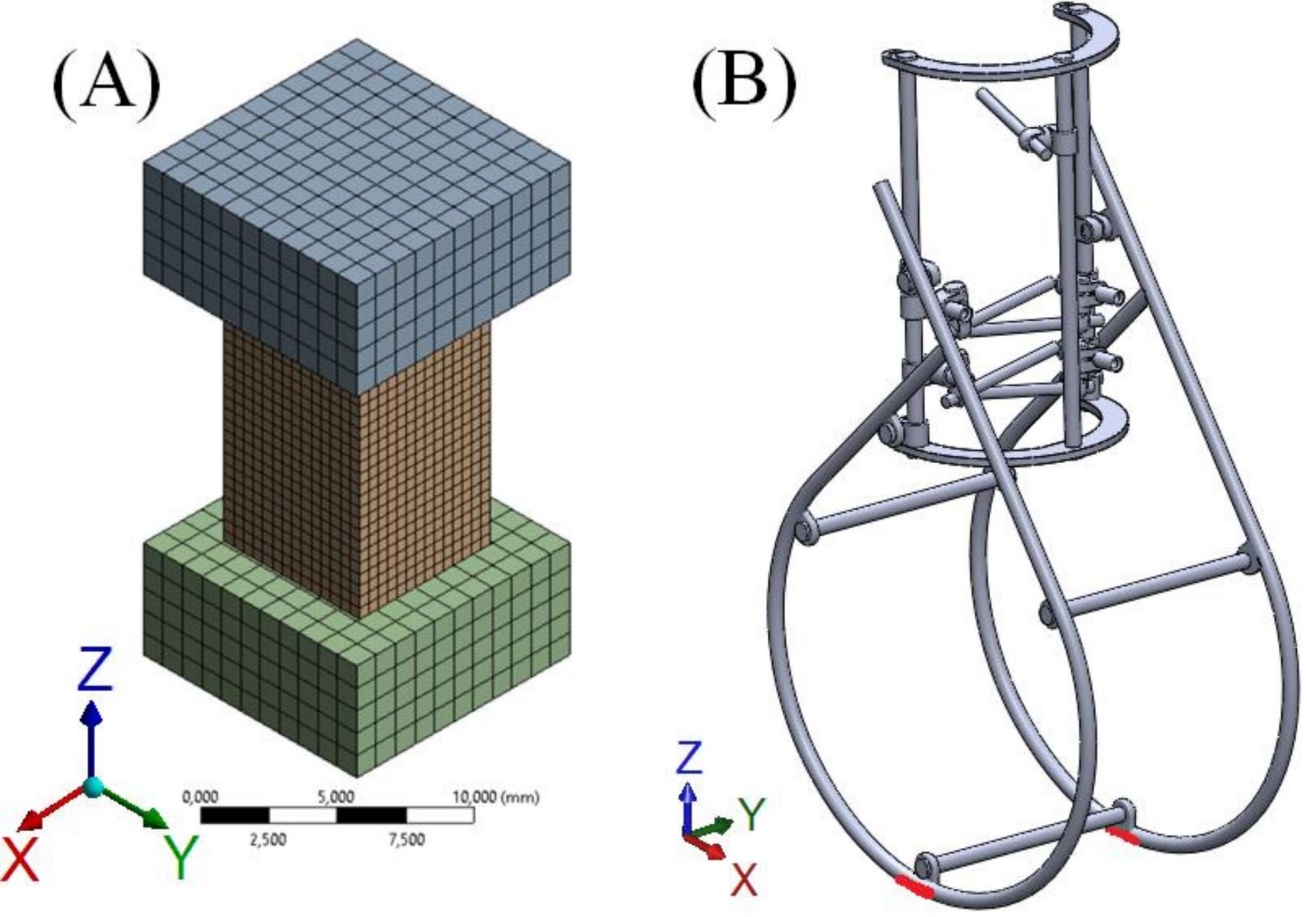
Model of (**A**) bone sample with machine handles and (**B**) external fixator used in simulations.

In the three–dimensional simulations of the uniaxial compression test, machine handles were modelled as an elastic continuum (the Young modulus $\:2\cdot\:{10}^{5}$ MPa, the Poisson ratio 0.3), and the bone samples were modelled using the formulated constitutive equation. Simulations were proceeded to the same level of force and at the same strain rate as in the corresponding experiments. The models of handles and bone samples were designed in SolidWorks 2023 (Dassault Systems, France), saved as a STEP file, and imported into ANSYS. The models of bone samples had the exact dimensions as those used in the experiments. In ANSYS, all models were meshed with hexahedron elements, for bone with dimensions 0.5 mm and handles 1 mm (Fig. 1A). The mesh study was conducted to find the optimal mesh density. The lower handle model was fixed at the bottom surface. As for the upper handle, only degrees of freedom in the X and Y directions were fixed to make the numerical calculations more stable. Contact between the models of bone and handles was set as frictionless, as the friction coefficient had a minimal impact on the results and noticeably increased the solution time.

### Design of the external fixator and simulations

DICOM files obtained from the CT scan were processed using MIMICS 14.0 (Materialise HQ, Belgium), producing models of cortical and trabecular bones of the MC III as STL files. The STL files of both structures were then converted into STEP files using SolidWorks 2023 Scan to 3D module.

The external fixator was designed by our team and modelled in SolidWorks 2023 (Fig. 1B). To simplify simulations, screws were modelled as cylinders of a diameter of 6 mm.

**Fig. 2:**
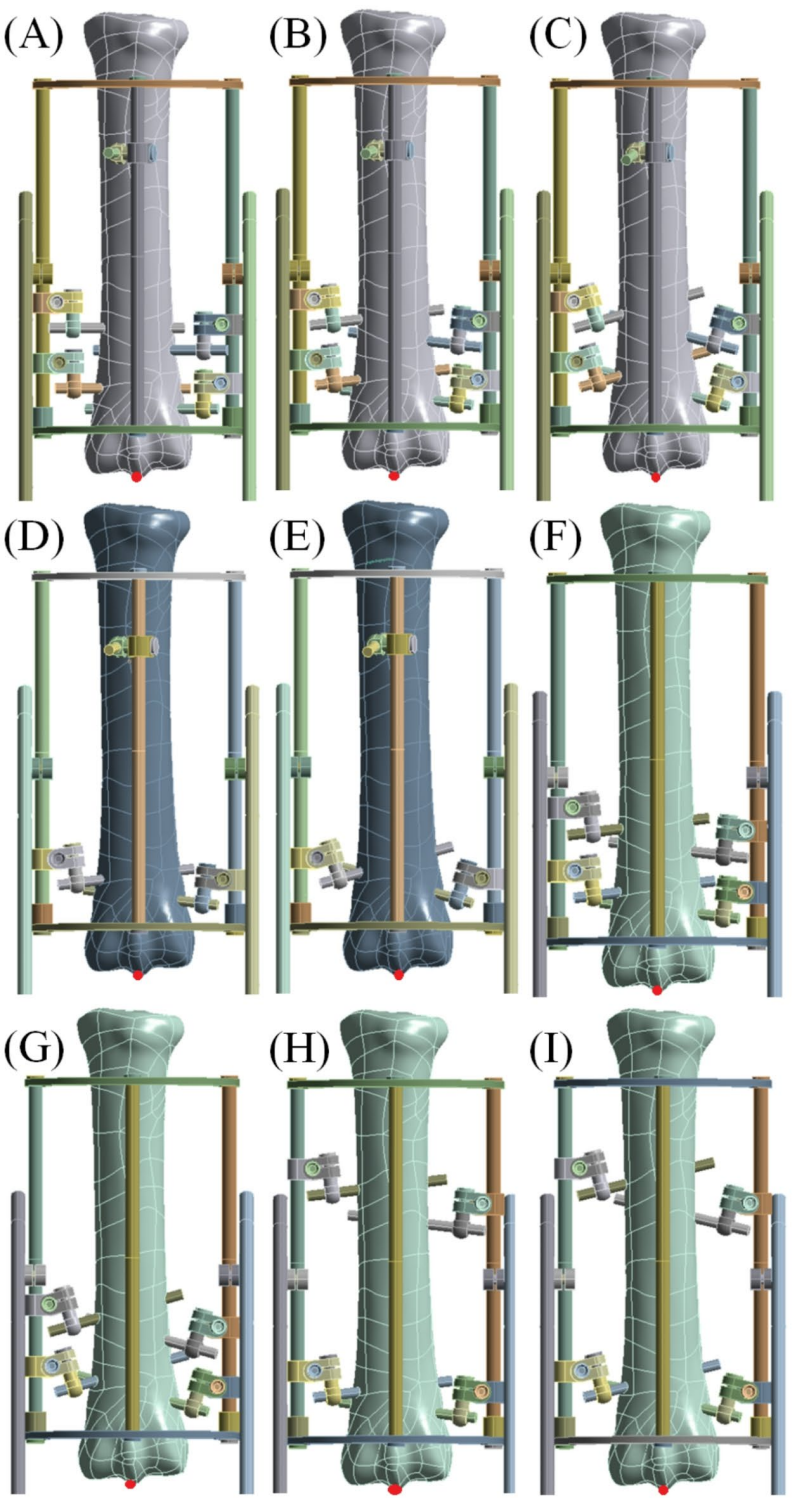
Nine configurations of the external fixator. (**A**) I – 1 screw in the diaphysis, 4 in the distal metaphysis, parallel to the horizontal plane; (**B**) II – 1 diaphysis screw, 4 distal screws at 7º; (**C**) III – 1 diaphysis screw, 4 distal screws at 14º; (**D**) IV – 1 diaphysis screw, 2 distal screws at 7º; (**E**) V – 1 diaphysis screw, 2 distal screws at 14º; (**F**) VI – 4 distal screws at 7º; (**G**) VII – 4 distal screws at 14º; (**H**) VIII – 2 diaphysis screws, 2 distal screws at 7º; (**I**) IX – 2 diaphysis screws, 2 distal screws at 14º.

Nine different configurations of screw alignment and various number of screws in the fixator based on previous studies^26,27^ were modelled as follows: I – one screw in the diaphysis, four screws in the distal metaphysis, screws parallel to the horizontal plane (Fig. 2A), II – one screw in the diaphysis, four screws in the distal metaphysis, screws 7º from the horizontal plane (Fig. 2B), III – one screw in the diaphysis, four screws in the distal metaphysis, screws 14º from the horizontal plane (Fig. 2C), IV – one screw in the diaphysis, two screws in the distal metaphysis, screws 7º from the horizontal plane (Fig. 2D), V – one screw in the diaphysis, two screws in the distal metaphysis, screws 14º from the horizontal plane (Fig. 2E), VI – four screws in the distal metaphysis, screws 7º from the horizontal plane (Fig. 2F), VII – four screws in the distal metaphysis, screws 14º from the horizontal plane (Fig. 2G), VIII – two screws in the diaphysis, two screws in the distal metaphysis, screws 7º from the horizontal plane (Fig. 2H), IX – two screws in the diaphysis, two screws in the distal metaphysis, screws 14º from the horizontal plane (Fig. 2I). All external fixators with MC III bone were saved as STEP files.

Simulations of the external fixator compression were proceeded in ANSYS 2021. The boundary conditions, material properties and mesh settings were the same in every configuration. All the external fixator parts were modelled as stainless steel (the Young modulus $\:2\cdot\:{10}^{5}$ MPa, the Poisson ratio 0.3). Trabecular and cortical bone tissues were modelled using the formulated constitutive equation with constants obtained during the calibration process. The external fixator, as described in detail in^26,27^, was attached to the MC III. From the fixation point, a vertically oriented metal frame extended into the hoop-shaped frame, transmitted loads from the ground. The hoop-shaped frame was in direct contact with the ground (red area in Fig. 1B). The force of 2743 N was applied vertically downwards to the proximal surface of the bone^26^. All parts of the fixator and bone models were meshed with tetrahedron elements. The mesh study was conducted to find the optimal mesh density. The number of elements in every configuration was around 200,000 elements. We used the Face sizing option on the faces with contact and on the faces where we assumed the stress concentrations would be and solved the simulation. Then we decreased the size of the elements in the Face sizing option and solved the simulation again. We repeated the procedure until the difference between the results of the simulations were within the given tolerance. Contact between potentially moving parts was set as frictional with Normal Lagrange formulation, with a friction coefficient of 0.74. Contact between screws and bone was set as frictional with Normal Lagrange formulation, with a friction coefficient of 0.9. Contact between parts and the screws that held the fixator together was set as bonded. In our study we wanted to check what happened with the fixator under the maximum force and to assess bone reaction to that load. Therefore, the simulation step was set to 1 s and was divided into 20 substeps to facilitate numerical calculations.

### Ethics declarations

Research using postmortem samples does not fall under legislation for the protection of animals used for scientific purposes, as outlined in the national decree-law (Dz. U. 2015 poz. 266) and the 2010/63/EU directive.

## Results

### Identification of constants

**Fig. 3:**
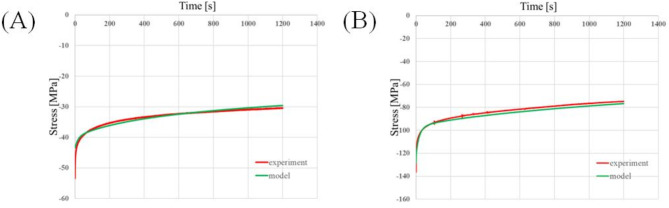
Model curve fitted to the stress relaxation results (**A**) trabecular bone, (**B**) cortical bone.

The relaxation times determine how quickly the material recovers from the applied load. During the relaxation test simulations, four relaxation times were needed to correctly imitate the behaviour of trabecular bone, whereas five were needed for cortical bone. Thus, the values of $\:n$ in Eq. (2) for both tissues were determined. The result of the curve–fitting process is presented in Fig. 3. The curves obtained from the stress relaxation tests are shown by the red solid line and those described by Eq. 10 are shown by green solid lines.

**Fig. 4:**
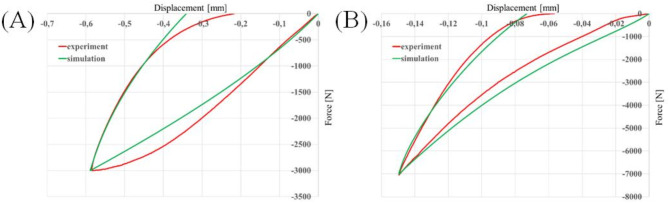
Curves from simulations fitted to the results from the uniaxial compression test for (**A**) trabecular bone and (**B**) cortical bone.

In Fig. 4, a graphical representation of the uniaxial compression test curve–fitting process is presented for trabecular bone (Fig. 4A) and cortical bone (Fig. 4B). Curves from the experiments are shown by a red solid line, while a green solid line represents curves described by the constitutive model and obtained from the simulations.

The behaviour of trabecular bone was best represented by the two–parameter Mooney Rivlin model, while the behaviour of cortical bone is best represented by the five–parameter Mooney Rivlin model. The identified constants for trabecular bone were $\:{c}_{10}=200\:\text{M}\text{P}\text{a}$, $\:{c}_{01}=105\:\text{M}\text{P}\text{a}$, $\:D=0.001$ MPa^−1^, $\:{g}_{1}=0.02$, $\:{g}_{2}=0.9$, $\:{g}_{3}=0.03$, $\:{g}_{4}=0.03$, $\:{\tau\:}_{1}=15\:s$, $\:{\tau\:}_{2}=28\:\text{s}$, $\:{\tau\:}_{3}=158\:\text{s}$, $\:{\tau\:}_{4}=1200\:\text{s}$ and for cortical bone: $\:{c}_{10}=1853\:\text{M}\text{P}\text{a}$, $\:{c}_{01}=-307\:\text{M}\text{P}\text{a}$, $\:{c}_{20}=1,760,513$ MPa, $\:{c}_{11}=2,168,232$ MPa, $\:{c}_{02}=-6528$ MPa, $\:D=0.00043$ MPa^−1^, $\:{g}_{1}=0.44$, $\:{g}_{2}=0.29$, $\:{g}_{3}=0.03$, $\:{g}_{4}=0.15$, $\:{g}_{5}=0.03\:$, $\:{\tau\:}_{1}=1\:\text{s}$, $\:{\tau\:}_{2}=10\:\text{s}$, $\:{\tau\:}_{3}=113\:\text{s}$, $\:{\tau\:}_{4}=1200\:\text{s}$, $\:{\tau\:}_{5}=1517$ s.

### Simulations

**Fig. 5:**
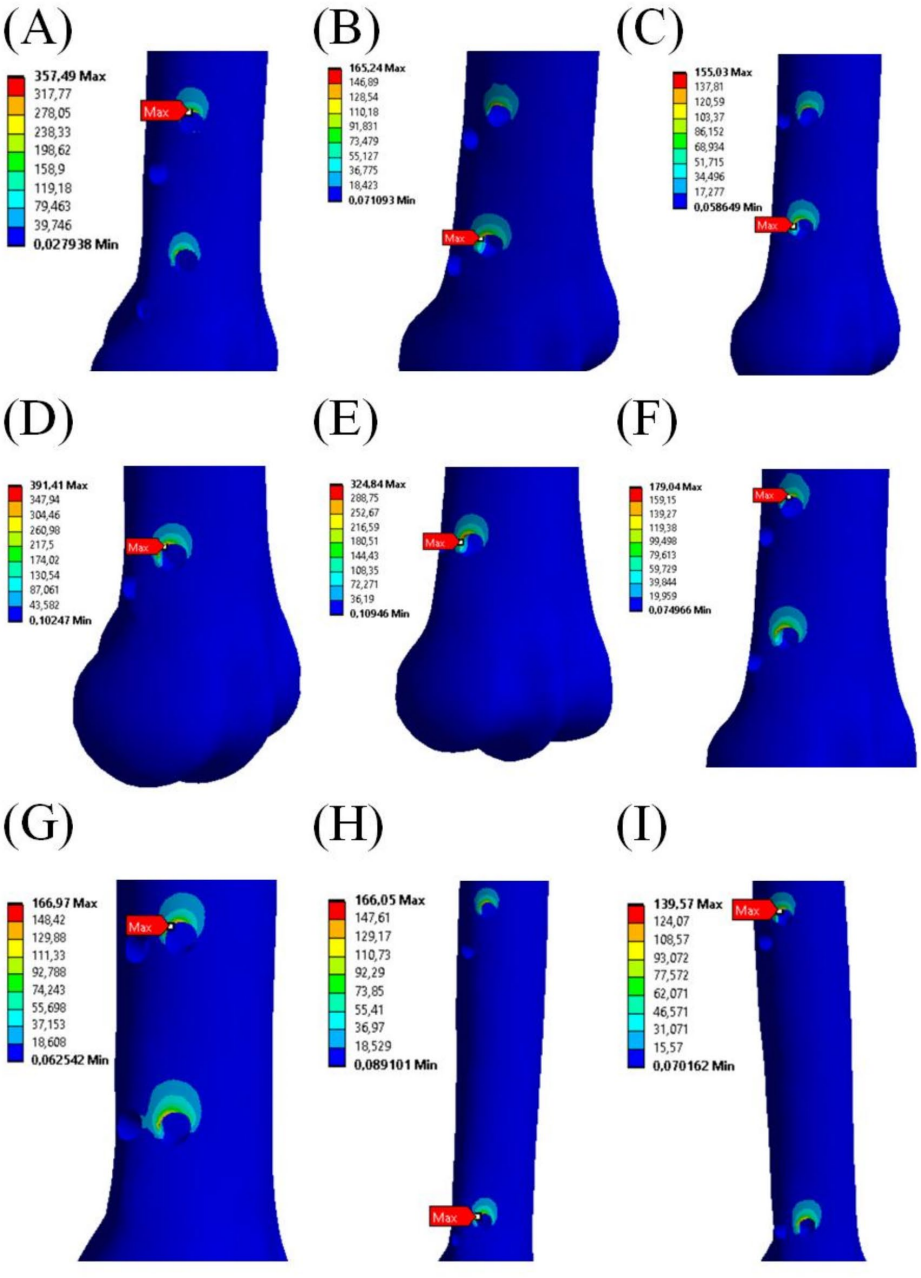
Positions and values of maximum von Mises stress in bone for each external fixator configuration. (**A**) I – 1 screw in the diaphysis, 4 in the distal metaphysis, parallel to the horizontal plane; (**B**) II – 1 diaphysis screw, 4 distal screws at 7º; (**C**) III – 1 diaphysis screw, 4 distal screws at 14º; (**D**) IV – 1 diaphysis screw, 2 distal screws at 7º; (**E**) V – 1 diaphysis screw, 2 distal screws at 14º; (**F**) VI – 4 distal screws at 7º; (**G**) VII – 4 distal screws at 14º; (**H**) VIII – 2 diaphysis screws, 2 distal screws at 7º; (**I**) IX – 2 diaphysis screws, 2 distal screws at 14º.

**Fig. 6:**
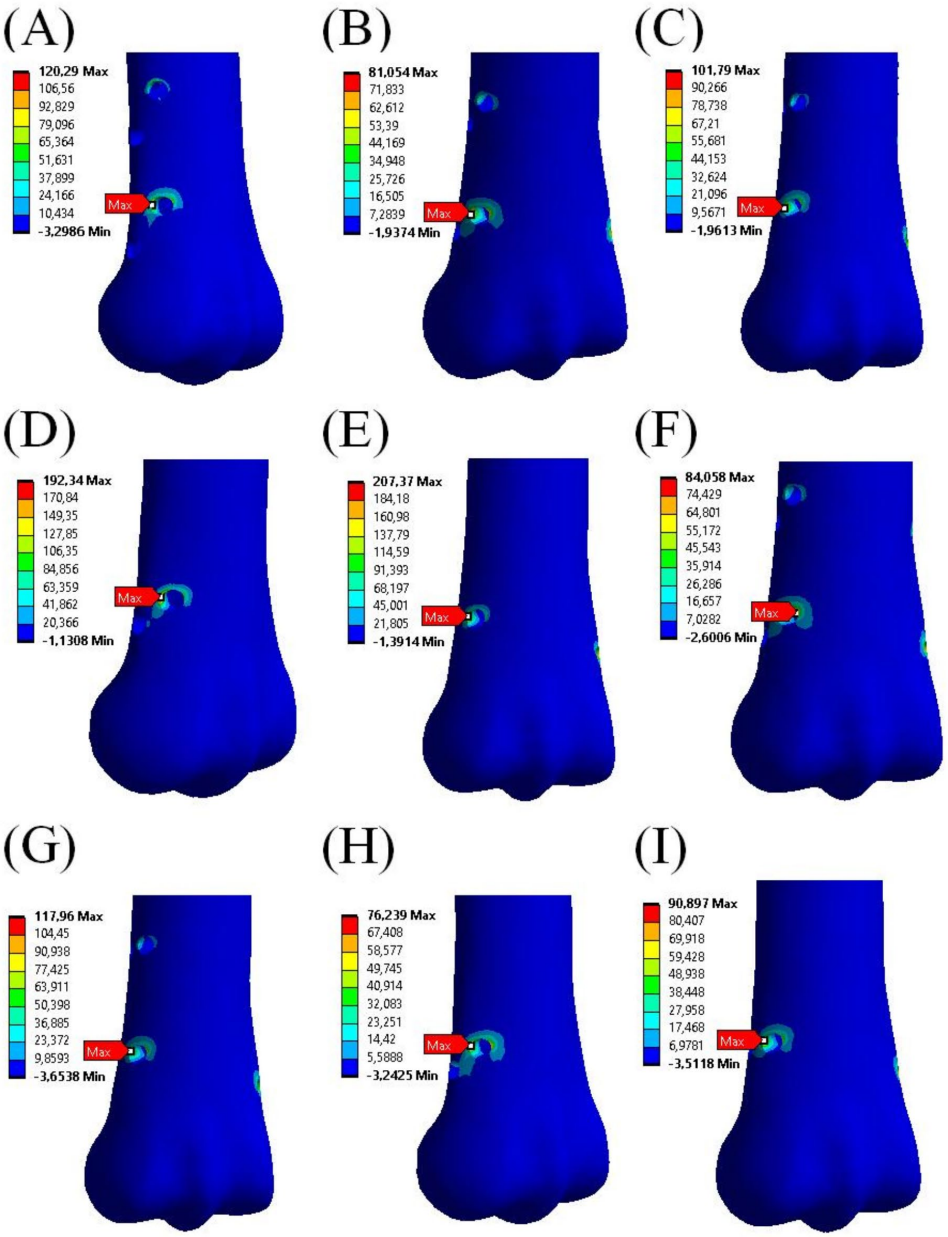
Positions and values of maximum principal stress in bone for each external fixator configuration. (**A**) I – 1 screw in the diaphysis, 4 in the distal metaphysis, parallel to the horizontal plane; (**B**) II – 1 diaphysis screw, 4 distal screws at 7º; (**C**) III – 1 diaphysis screw, 4 distal screws at 14º; (**D**) IV – 1 diaphysis screw, 2 distal screws at 7º; (**E**) V – 1 diaphysis screw, 2 distal screws at 14º; (**F**) VI – 4 distal screws at 7º; (**G**) VII – 4 distal screws at 14º; (**H**) VIII – 2 diaphysis screws, 2 distal screws at 7º; (**I**) IX – 2 diaphysis screws, 2 distal screws at 14º.

Four criteria were selected to evaluate configurations of the external fixator: von Mises stress and maximum principal stress in the cortical and trabecular bone, von Mises strain in the cortical and trabecular bone, von Mises stress in the screws and displacement of the point in MC III bone in the vertical axis. The most important criterion was von Mises stress and maximum principal stress in the cortical bone. Too much stress in the bone can cause osteonecrosis^36,37^. In Fig. 5, von Mises stress distribution and values of maximum stress in cortical bone in every configuration of the external fixator are presented. Figure 6 shows distribution of maximum principal stress in the bone issue.

In all the cases, the maximum von Mises stress value was in the areas of the screw holes. For cortical bone, the lowest value of maximum stress, 139.57 MPa, was in configuration IX near the highest screw in the bone diaphysis. The second lowest value, 155.03 MPa, was in configuration III in the lowest left screw in the distal metaphysis. The highest value was in configuration IV, 391.41 MPa, near one of the two screws in the distal metaphysis. For trabecular bone, the lowest value of maximum stress, 6.43 MPa, was in configuration IX. The second lowest value, 155.03 MPa, was in configuration I. The highest value was in configuration IV, 30.22 MPa. The values of the maximum stress in the cortical and trabecular bone in every configuration are shown in Table 1.

**Table 1:** The values of maximum von Mises stress in the cortical and trabecular bone in every configuration.

Config.	I	II	III	IV	V	VI	VII	VIII	IX
Cortical bone									
Max stress [MPa]	357.49	165.24	155.03	391.41	324.84	179.04	166.97	166.5	139.57
Trabecular bone									
Max stress [MPa]	6.77	12.83	8.51	30.22	11.31	11.89	7.72	16.52	6.43

Max stress = maximum von Mises stress, Config. = configuration of screws as following: I – 1 screw in the diaphysis, 4 in the distal metaphysis, parallel to the horizontal plane; (B) II – 1 diaphysis screw, 4 distal screws at 7º; (C) III – 1 diaphysis screw, 4 distal screws at 14º; (D) IV – 1 diaphysis screw, 2 distal screws at 7º; (E) V – 1 diaphysis screw, 2 distal screws at 14º; (F) VI – 4 distal screws at 7º; (G) VII – 4 distal screws at 14º; (H) VIII – 2 diaphysis screws, 2 distal screws at 7º; (I) IX – 2 diaphysis screws, 2 distal screws at 14º.

The maximum principal stress in all the configurations also located in the close vicinity of the screw holes. It can be noted that the lowest value of the maximum principal stress, 76.24 MPa, is observed in configuration VIII near the lower screw in the metaphysis. Also, in the case of configurations II and VI the maximum principal stress is relatively low, i.e. 81.05 MPa and 84.06 MPa, respectively. The highest value of the maximum principal stress, 207.37 MPa, was for configuration V. All those values were in the area of the lower screw in the metaphysis. Table 2 summarises the maximum principal stress in cortical and trabecular bone for all the configurations.

The highest values of von Mieses strain in the cortical bone in every configuration were in the same areas as the maximum stress values. The values of the maximum strain are presented in Table 3. For cortical bone, the lowest value of maximum strain was in configuration IX, and it was 0.0085. The second lowest was in configuration III, 0.009. The highest value of maximum strain was in configuration IV, 0.0133. For trabecular bone the lowest value of maximum strain was in configuration IX, and it was 0.0036. The second lowest was in configuration I, 0.0039. The highest value of maximum strain was in configuration IV, 0.0170.

**Table 2:** The values of maximum principal stress in the cortical and trabecular bone in every configuration.

Config.	I	II	III	IV	V	VI	VII	VIII	IX
Cortical bone									
Max stress [MPa]	120.29	81.05	101.79	192.34	207.37	84.06	117.96	76.24	90.90
Trabecular bone									
Max stress [MPa]	5.60	8.46	6.35	15.71	8.72	8.42	5.84	9.08	6.17

Max stress = maximum principal stress, Config. = configuration of screws as following: I – 1 screw in the diaphysis, 4 in the distal metaphysis, parallel to the horizontal plane; (B) II – 1 diaphysis screw, 4 distal screws at 7º; (C) III – 1 diaphysis screw, 4 distal screws at 14º; (D) IV – 1 diaphysis screw, 2 distal screws at 7º; (E) V – 1 diaphysis screw, 2 distal screws at 14º; (F) VI – 4 distal screws at 7º; (G) VII – 4 distal screws at 14º; (H) VIII – 2 diaphysis screws, 2 distal screws at 7º; (I) IX – 2 diaphysis screws, 2 distal screws at 14º.

**Table 3:** The values of maximum von Mises strain in the cortical and trabecular bone in every configuration.

Config.	I	II	III	IV	V	VI	VII	VIII	IX
Cortical bone									
Max strain [-]	0.0128	0.0092	0.0090	0.0133	0.0124	0.0095	0.0092	0.0092	0.0085
Trabecular bone									
Max strain [-]	0.0039	0.0075	0.0047	0.0170	0.0063	0.0070	0.0043	0.0093	0.0036

Max strain = maximum von Mises strain, Config. = configuration of screws as following: I – 1 screw in the diaphysis, 4 in the distal metaphysis, parallel to the horizontal plane; (B) II – 1 diaphysis screw, 4 distal screws at 7º; (C) III – 1 diaphysis screw, 4 distal screws at 14º; (D) IV – 1 diaphysis screw, 2 distal screws at 7º; (E) V – 1 diaphysis screw, 2 distal screws at 14º; (F) VI – 4 distal screws at 7º; (G) VII – 4 distal screws at 14º; (H) VIII – 2 diaphysis screws, 2 distal screws at 7º; (I) IX – 2 diaphysis screws, 2 distal screws at 14º.

The highest values of the maximum principal strain in cortical bone were reported in configuration IV and V. The lowest values were in configurations II, VI, VIII and IX. In trabecular bone the highest maximum principal strain values were in configuration IV, the lowest – in configurations I, VII and IX. The values of the maximum principal strain are presented in Table 4.

**Table 4:** The values of maximum principal strain in the cortical and trabecular bone in every configuration.

Config.	I	II	III	IV	V	VI	VII	VIII	IX
Cortical bone									
Max strain [-]	0.010	0.008	0.009	0.013	0.013	0.008	0.010	0.007	0.008
Trabecular bone									
Max strain [-]	0.003	0.006	0.004	0.014	0.005	0.006	0.003	0.008	0.003

Max strain = maximum principal strain, Config. = configuration of screws as following: I – 1 screw in the diaphysis, 4 in the distal metaphysis, parallel to the horizontal plane; (B) II – 1 diaphysis screw, 4 distal screws at 7º; (C) III – 1 diaphysis screw, 4 distal screws at 14º; (D) IV – 1 diaphysis screw, 2 distal screws at 7º; (E) V – 1 diaphysis screw, 2 distal screws at 14º; (F) VI – 4 distal screws at 7º; (G) VII – 4 distal screws at 14º; (H) VIII – 2 diaphysis screws, 2 distal screws at 7º; (I) IX – 2 diaphysis screws, 2 distal screws at 14º.

**Fig. 7:**
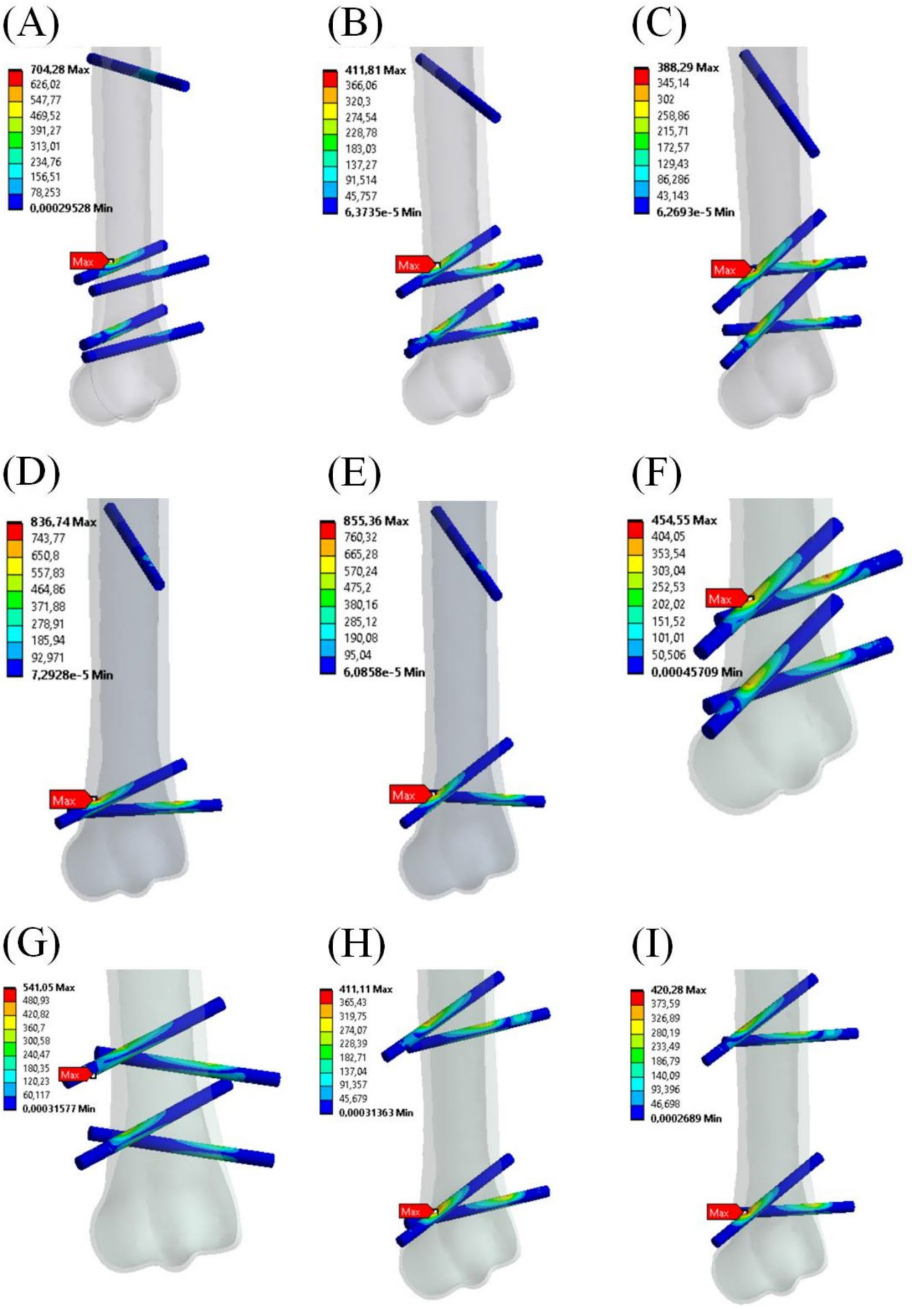
Positions and values of maximum von Mises stress in screws for each external fixator configuration. (**A**) I – 1 screw in the diaphysis, 4 in the distal metaphysis, parallel to the horizontal plane; (**B**) II – 1 diaphysis screw, 4 distal screws at 7º; (**C**) III – 1 diaphysis screw, 4 distal screws at 14º; (**D**) IV – 1 diaphysis screw, 2 distal screws at 7º; (**E**) V – 1 diaphysis screw, 2 distal screws at 14º; (**F**) VI – 4 distal screws at 7º; (**G**) VII – 4 distal screws at 14º; (**H**) VIII – 2 diaphysis screws, 2 distal screws at 7º; (**I**) IX – 2 diaphysis screws, 2 distal screws at 14º.

In Fig. 7, the stress distribution and values of maximum von Mises stress in all screws in every configuration of the external fixator are presented. In all cases except configuration VII, the maximum stress value was on the top surface of one of the screws. In configuration VII, the maximum value was on the bottom surface of the highest screw in the distal metaphysis. The lowest value of maximum stress was in configuration III in the highest screw in the distal metaphysis, and it was 388.29 MPa. The second lowest value of maximum stress was in configuration VIII and was in one of the two screws in the distal metaphysis, 411.11 MPa. The highest value of maximum stress was in configuration V, also in one of the two screws in the distal metaphysis, and it was 855.36 MPa.

The values of the maximum von Mises stress in all screws in every configuration are shown in Table 5.

**Table 5:** The values of maximum von Mises stress in the screws in every configuration.

Config.	I	II	III	IV	V	VI	VII	VIII	IX
Max stress [MPa]	704.28	411.81	388.29	836.74	855.36	454.55	541.05	411.11	420.28

Max stress = maximum von Mises stress, Config. = configuration of screws as following: I – 1 screw in the diaphysis, 4 in the distal metaphysis, parallel to the horizontal plane; (B) II – 1 diaphysis screw, 4 distal screws at 7º; (C) III – 1 diaphysis screw, 4 distal screws at 14º; (D) IV – 1 diaphysis screw, 2 distal screws at 7º; (E) V – 1 diaphysis screw, 2 distal screws at 14º; (F) VI – 4 distal screws at 7º; (G) VII – 4 distal screws at 14º; (H) VIII – 2 diaphysis screws, 2 distal screws at 7º; (I) IX – 2 diaphysis screws, 2 distal screws at 14º.

The point whose displacement was measured was located in the lower articulation surface of the MC III bone model (red dot in Fig. 2). The bone displacement was the lowest in configuration IV, and the displacement value was 9.18 mm. The bone had the least displacement in configuration VII, the value was 8.50 mm. Slightly larger displacements, 8.52 mm and 8.54 mm, were in configurations VI and III, respectively. The displacement values for each configuration of the external fixator are presented in Table 6.

**Table 6:** The values of maximum displacement of the point on the bone in every configuration.

Config.	I	II	III	IV	V	VI	VII	VIII	IX
Bone displacement [mm]	-8.92	-8.57	-8.54	-9.18	-9.16	-8.52	-8.50	-8.63	-8.63

Config. = configuration of screws as following: I – 1 screw in the diaphysis, 4 in the distal metaphysis, parallel to the horizontal plane; (B) II – 1 diaphysis screw, 4 distal screws at 7º; (C) III – 1 diaphysis screw, 4 distal screws at 14º; (D) IV – 1 diaphysis screw, 2 distal screws at 7º; (E) V – 1 diaphysis screw, 2 distal screws at 14º; (F) VI – 4 distal screws at 7º; (G) VII – 4 distal screws at 14º; (H) VIII – 2 diaphysis screws, 2 distal screws at 7º; (I) IX – 2 diaphysis screws, 2 distal screws at 14º.

## Discussion

The first research on the use of an external orthopaedic device was presented by Kirk in 1952^38^. Further developments were proposed in 1986 with a device that allowed limb loading immediately after surgery^39^. In 1991, Nemeth and Back reported the results of treating 52 horses with a walking cast – a specific external fixator^40^. Research on external fixators has continued^26,27,41–43^, showing promising clinical results for treating fractures in horses, but also highlighting the need for broader recognition and further optimization of these devices. This study addresses the indicated need by developing the mathematical and numerical modelling of the bone–screws interactions when an external fixator for P1 fracture treatment is applied. The study presents a computational modelling of a novel orthopaedic device for treating complex and comminuted P1 fractures in horses, considering various configurations for stabilizer fixation to the MC III.

Finite element analyses were conducted to determine the favourable screw configuration. Finite element analyses have been already used in equine orthopaedics to model the MC II–IV with comminuted diaphyseal fractures^44^ as well as to determine the effect of six transcortical pin parameters on bone–pin interface stresses in the MC III^45^. This numerical model included the device, screws, and MC III, with bone tissue modelled as a viscoelastic medium, a common approach in finite element simulations involving bone^31,46^. This method allows for predicting tissue response to load transferred through the screws and provides an initial assessment of the bone healing process^47^. Apex screws, 6 mm in diameter, were used in the study, as this size minimizes bone damage during surgery and provide good strength^48^. The numerical simulations considered three angles relative to a horizontal plane: 0^o^, 7^o^ and 14^o^, as higher angles were deemed disadvantageous for bone response.

The results of our numerical simulations indicate that the deformation of the external fixator, measured by the vertical displacement of the bone, was lowest in configuration VII. However, configurations II, III and VI showed similarly low displacement values (Table 6). The highest deformation occurred in configurations IV and V, where only two screws were used in the distal metaphysis. Also, the highest von Mises stress in the screw system was observed in configurations IV and V (Table 5), with stress values close to or exceeding the yield strength of the material used for screws^49,50^. These findings suggest that two distal screws and one proximal screw may not provide adequate stability or functionality for the external fixator. The lowest von Mises stress values in cortical bone (Fig. 5; Table 1) were recorded in configurations II, III, VI, VII, VIII, and IX, indicating that four screws are necessary for stable fixation, with a possible fifth screw in the proximal part bone (as in configurations II and III). The same conclusion can be drawn from maximum principal stress distribution shown in Fig. 6 where the maximal values of the stress were noticed for configuration IV and V. Table 2 confirms that observation. The highest stress value in trabecular bone was reported in configuration IV. This concerns both von Mises stress and maximum principal stress (Tables 1 and 2). The stress concentration in trabecular bone for configuration V was on the similar level as that in the remaining configurations. Thus, from that point of view configuration V seems to be comparable to the other ones. One may note that previous studies on intra–axial positioning in the MC III investigated configurations involving one to six screws^44,45^. However, in both studies^44,45^, the optimal screw configuration was not directly indicated as well as the external fixator was not considered as an additional transferring device contributing to weight–bearing by the injured limb^15,16^.

Analysis of von Mises stress values in cortical bone leads to a conclusion that configurations I, IV and V should not be applied in P1 fracture treatment process as those values are much higher than the reported in literature yield stress for equine cortical bone, i.e. 148 MPa in tension and 146 ÷ 185 MPa^51^ or 192 MPa^52^ in compression. In this regard, configuration IX seems to be the most adequate. It seems, however, that configurations II, III, VII and VIII could be possibly applied. This observation is confirmed by the maximum principal stress concentrations in cortical bone. Configurations IV and V should be discarded from the consideration. The values of maximum principal stress in the remaining configurations are lower than the yield stress of equine cortical bone. Also, the values of von Mises strain and maximum principal strain for those configurations (Tables 3 and 4) are below the yield strain which is in the range of 0.01 ÷ 0.0176 ^51,52^.

Although we conducted static analyses in which the load acting on the model was constant, we discuss our results in terms of fatigue behaviour of MC III. In this regard, some configurations give undesirable performance. Comparing the maximum principal stress values to fatigue strength of MC III obtained at approx. 50,000 cycles (100 MPa^53^) we conclude that configurations II, VI, VIII and IX gave satisfactory results. It seems that also stabiliser in configuration III might be used to treat the P1 fracture as the maximum principal stress in this case equals 101.79 MPa. It must be noticed that fatigue behaviour of MC III was determined in bending tests. Zioupos et al. determined compressive fatigue strength of human cortical bone^54^. According to their results it is equal 150 MPa. If we assume that equine cortical bone fatigue strength is at least of that value, the range of applicable configurations is extended by III and VII.

In this study, we also focused on simulating the bone tissues’ reaction to the stabilizer to predict its behaviour. Bone, both trabecular and cortical, was modelled as a viscoelastic continuum, a widely used approach in bone tissue simulations^55–57^. The results of numerical analyses showed that the highest stress values in the bone tissues occurred in configurations I, IV and V. Similarly, these screw orientations resulted in the highest strain in the bone near the holes (Table 2), which is undesirable as it could lead to excessive osteonecrosis^36,37^. Conversely, too low stress can cause bone atrophy, leading to implant loosening and destabilization^44^.

Therefore, configurations II, III, VI, VII, VIII, and IX appear to be the most beneficial from the standpoint bone response. However, based on our clinical practice and experience, the four lower screws should be implanted in the distal metaphysis, which contains sufficient bone tissues. This excludes configurations VIII and IX. Additionally, the stress values and distribution in the screws (as shown in Fig. 7; Table 5) indicate that the fifth screw in the proximal part of the bone contributes to more favourable biomechanical conditions. This finding eliminates configuration VI and VII. Thus, configurations II and III are concluded to be the most suitable for P1 bone fracture treatment.

The maximum von Mises stress in the orthopaedical screws (Fig. 7; Table 5) are higher than the reported yield strength for stainless steel, i.e. 484 MPa^58^, for configurations I, IV, V and VII. However, other studies^59^ show that the value of 0.2% proof strength for full-thread cortical screws made of stainless steel is 876 MPa. Li et al. reported that the yield strength of the orthopaedical steel is in the range of 250 ÷ 900 MPa^60^. This makes all the configurations acceptable in terms of the screws’ strength.

Research on fatigue behaviour of stainless steel show that fatigue strength is in the range of 160 ÷ 400 MPa which corresponds to the fatigue life > 10^6^ and 1000 cycles, respectively^61,62^. The maximum values of von Mises stress in the screws obtained in our study are higher than fatigue strength of the steel for most of the configurations. Only the stress value for configuration III is lower than 400 MPa (388,29 MPa). For configurations II and VIII the stress values are somewhat higher (411 MPa). Those results show that screws of higher diameter should be used to fix the stabiliser.

Among, the limitations of this study one may highlighted that the bone–stabiliser system was simulated under one type of load, i.e. vertical constant load on the bone. However, the primary aim of this study was to introduce a concept of a new stabiliser construction and to verify its functionality through numerical simulations. Despite using a single load type, the results provide valuable insights into how the device should be constructed. Identifying favourable screw combinations, including the angle and number of screws, that minimize stresses and strains at the bone–screws interface, should reduce the risk of both acute and chronic failure^45^ and enhance the safety and effectiveness of external fixator in treating comminuted P1 fractures during clinical use.

Despite this limitation, the main strength and advantage of the simplified modelling approach is its ability to make multiple comparisons across different screws number and angles. These findings provide a foundation for investigating additional aspects of the external fixator that may influence stress at the bone–screws interface, through further ex vivo and in vivo research. Additional ex vivo studies are required to test the maximum load that these fixator configurations can bear, considering the minimal and maximal weight–bearing of the horse’s limb^15,16^ to support the standing post–surgery^26,27^.

Furthermore, bone remodelling, which depends heavily on mechanical stimuli like stress and strain^63,64^, was not included in the simulations. These factors are crucial in the external fixator design process and should be incorporated into the next ex vivo stage of the study. Lastly, although we explored certain screw configurations, other combinations could be considered. For this preliminary study, we selected screw configurations that appeared most clinically feasible based on previous studies^26,27,44,45^.

In our study we used screws inserted into one layer of cortical bone and into trabecular bone. It might, however, be interesting to apply screws placed in both layers of cortical bone, i.e. through the bone width. Such an approach would influence bone remodeling phenomenon and, consequently, stability of the fixator.

## Conclusions

The study successfully develops the mathematical and numerical modelling of the bone–screws interactions when an external fixator for P1 fracture treatment is applied. From biomechanical and clinical point of view, the most favourable configuration of screws used to attach an external fixator to MC III is configuration II (1 diaphysis screw, 4 distal screws at 7º) and III (1 diaphysis screw, 4 distal screws at 14º). However, further ex vivo research is needed to test the maximal load that these fixator configurations can bear, before clinical use will follow up.

## Data Availability

The datasets generated during and/or analyzed during the current study are available from the corresponding author on reasonable request.
